# The GC-Rich Mitochondrial and Plastid Genomes of the Green Alga *Coccomyxa* Give Insight into the Evolution of Organelle DNA Nucleotide Landscape

**DOI:** 10.1371/journal.pone.0023624

**Published:** 2011-08-26

**Authors:** David Roy Smith, Fabien Burki, Takashi Yamada, Jane Grimwood, Igor V. Grigoriev, James L. Van Etten, Patrick J. Keeling

**Affiliations:** 1 Department of Botany, Canadian Institute for Advanced Research, University of British Columbia, Vancouver, British Columbia, Canada; 2 Department of Molecular Biotechnology, Graduate School of Advanced Sciences of Matter, Hiroshima University, Higashi-Hiroshima, Japan; 3 HudsonAlpha-JGI, HudsonAlpha Genome Sequencing Center, Huntsville, Alabama, United States of America; 4 Department of Energy Joint Genome Institute, Walnut Creek, California, United States of America; 5 Department of Plant Pathology and Nebraska Center for Virology, University of Nebraska, Lincoln, Nebraska, United States of America; French National Centre for Scientific Research, Université Paris-Sud, France

## Abstract

Most of the available mitochondrial and plastid genome sequences are biased towards adenine and thymine (AT) over guanine and cytosine (GC). Examples of GC-rich organelle DNAs are limited to a small but eclectic list of species, including certain green algae. Here, to gain insight in the evolution of organelle nucleotide landscape, we present the GC-rich mitochondrial and plastid DNAs from the trebouxiophyte green alga *Coccomyxa* sp. C-169. We compare these sequences with other GC-rich organelle DNAs and argue that the forces biasing them towards G and C are nonadaptive and linked to the metabolic and/or life history features of this species. The *Coccomyxa* organelle genomes are also used for phylogenetic analyses, which highlight the complexities in trying to resolve the interrelationships among the core chlorophyte green algae, but ultimately favour a sister relationship between the Ulvophyceae and Chlorophyceae, with the Trebouxiophyceae branching at the base of the chlorophyte crown.

## Introduction

Some of the most diverse and unusual mitochondrial and plastid DNAs (mtDNAs and ptDNAs) from all eukaryotes come from the Chlorophyta — a lineage comprising the majority of known green algal species [1]. Both among and within the four main chlorophyte classes — Prasinophyceae, Trebouxiophyceae, Ulvophyceae, and Chlorophyceae — there is an impressive range of mitochondrial and plastid genome sizes, gene complements, and noncoding DNA compositions (see [2] for a compilation). Moreover, the Chlorophyceae boasts some of the few documented cases of species with guanine- and cytosine-rich (GC-rich) mtDNA, which is a significant feat when considering that most mitochondrial (and plastid) genomes are highly biased in adenine and thymine (AT). The nonphotosynthetic chlorophycean *Polytomella capuana* has one of the highest recorded mtDNA GC contents (57%) [3], and analyses of the mtDNA-encoded *cox1* and *cob* from the chlorophyceans *Lobochlamys segnis* and *Lobochlamys culleus* revealed average GC compositions of 54 and 62%, respectively [4]. The only other published examples of GC-rich mitochondrial genomes (as of March 2011) come from the parasitic fungus *Candida subhashii* [5], a few teleost species ([6], and references therein), and the lycophyte *Selaginella moellendorffii* [7], [8]. The latter and other members of the *Selaginella* genus are also exceptional in that they are the only species known to contain GC-rich plastid genomes [7], [9].

The forces biasing organelle genomes towards G and C may differ among lineages. For instance, within the *P. capuana* mtDNA, the GC content is highest at silent sites, such as noncoding and third codon position, suggesting a neutral underpinning to the GC richness [3]. Conversely, for the *S. moellendorffii* mitochondrial and plastid genomes, the levels of G and C are highest at the more functionally constrained sites, like rRNA-coding regions and first and second codon positions, implying that the GC bias is driven by natural selection; complicating this interpretation, however, is the fact that many of the cytosine residues within the *Selaginella* mtDNA and ptDNA are post-transcriptionally edited to uracil [7]–[9]. Unraveling the mechanism responsible for the GC enrichment of organelle genomes might in turn help explain the near-ubiquity of AT-rich mitochondrial and plastid DNAs throughout the eukaryotic domain, a feature that is poorly understood but probably linked to AT mutation pressure [10], [11].

*Coccomyxa* sp. C-169 (formerly referred to as *Chlorella* sp. C-169; hereafter *Coccomyxa*) is a unicellular, free-living trebouxiophyte [12] whose nuclear genome is being sequenced by the United States Department of Energy Joint Genome Institute (DOE JGI). Our cursory scan of the *Coccomyxa* sequencing reads revealed elevated levels of G and C in what appeared to be mtDNA and ptDNA sequences, suggesting that complete assemblies of the *Coccomyxa* mitochondrial and plastid genomes may reveal a novel set of GC-rich organelle DNAs.

Moreover, there is currently a lack of trebouxiophyte organelle genome data, which limits the utility of the available green algal organelle genomes for phylogenetic analyses. This is significant because, although there is strong support for the Prasinophyceae forming a paraphyletic assemblage at the base of the Chlorophyta, the branching order of the Ulvophyceae, Trebouxiophyceae, and Chlorophyceae (UTC) remains unresolved and controversial [1], [13]. This is likely due to poor sampling and a lack of molecular sequence data from these groups, but it also reflects their antiquity, morphological diversity, and the potentially short timeframe over which they diverged from each other [13]–[15]. Depending on the ultrastructural characteristics and molecular data being used, all three possible branching orders of the UTC classes have been hypothesized [16]–[18]. Plastid genome multi-gene phylogenies have generally supported a sister relationship between the Ulvophyceae and Trebouxiophyceae [16], [18], whereas mtDNA phylogenies have tended to group the ulvophytes close to the chlorophyceans [19], [20]. These analyses, however, have been hindered because the only complete sets of trebouxiophyte organelle genomes come from *Pedinomonas minor* [18], [21] — a species that some have argued is not a trebouxiophyte at all, and whose mitochondrial genome shows high rates of evolution [20], [22], [23] — and the nonphotosynthetic parasite *Helicosporidium* sp. ATCC 50920 [20], [24] (hereafter *Helicosporidium*), whose plastid genome contains no genes for proteins that function in photosynthesis and is therefore poorly suited for multi-gene ptDNA phylogenies.

Here, we present the mitochondrial and plastid genome sequences from *Coccomyxa*. We investigate the architectures and nucleotide landscapes of these genomes as well as their potential for resolving the relationships among the crown chlorophytes.

## Results and Discussion

### Architecture of the Coccomyxa organelle genomes

**Figure 1:**
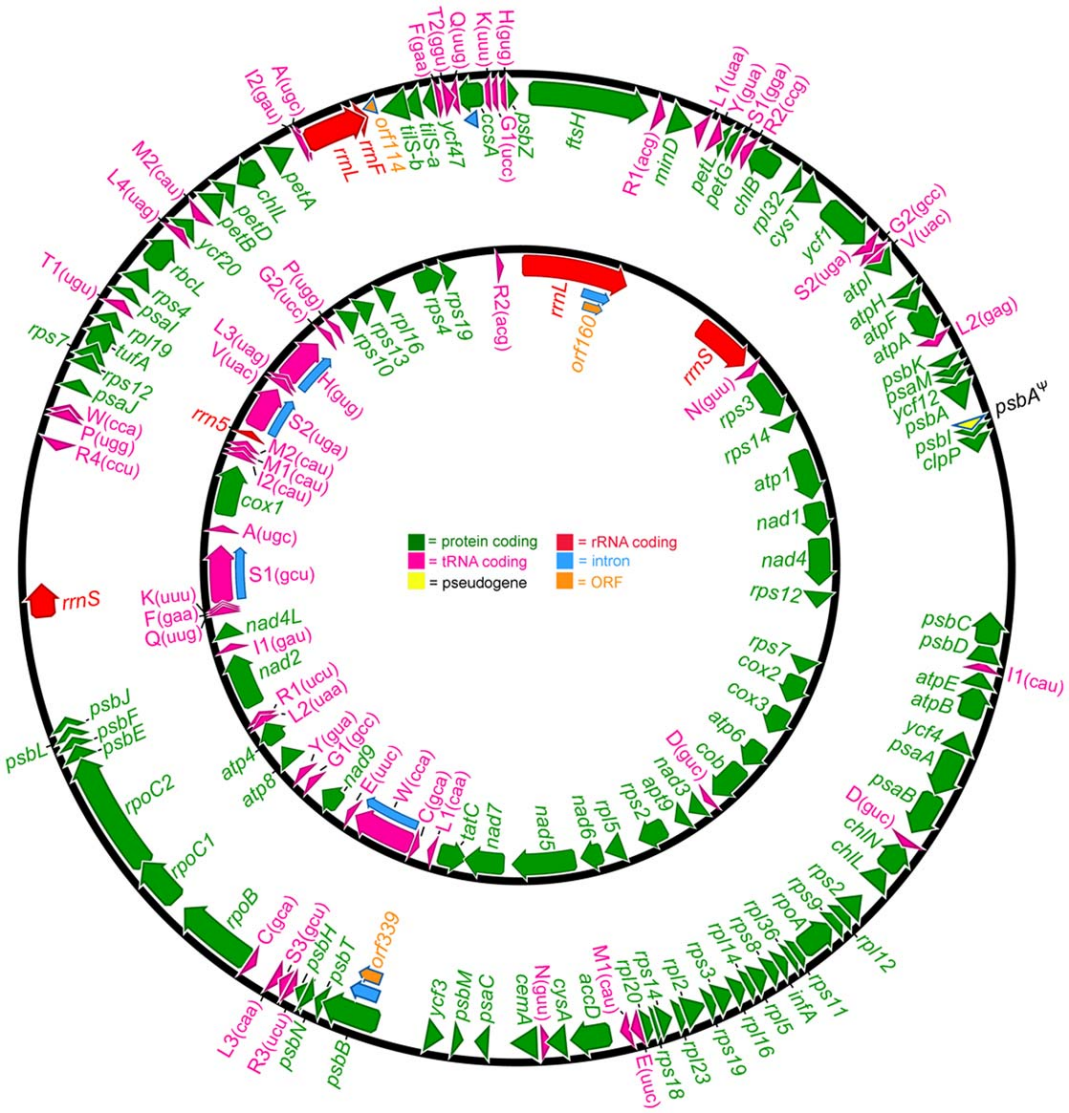
Genetic maps of the *Coccomyxa* plastid (outer) and mitochondrial (inner) genomes. The ptDNA and mtDNA sequences have respective lengths of 175.7 and 65.4 kb, and are deposited in GenBank under accession numbers HQ693844 and HQ874522. Arrows within the coding regions denote transcriptional polarities. Transfer RNA-coding regions are designated by the single-letter abbreviation of the amino acid they specify; their anticodon sequences are shown in brackets.

The *Coccomyxa* mitochondrial and plastid genome sequences (GenBank under accession numbers HQ874522 and HQ693844, respectively) are both circular-mapping [25], [26] (Figure 1) and, as predicted, are rich in G and C. These are the third set of trebouxiophyte mtDNA and ptDNA sequences described thus far; the other two, as mentioned above, come from *Helicosporidium* and *P. minor* [18], [20], [21], [24], and there is also complete sequence data for the ptDNAs of the trebouxiophytes *Chlorella vulgaris*, *Parachlorella kessleri*, and *Leptosira terrestris* [18], [27] and for the mtDNA of the nonphotosynthetic parasite *Prototheca wickerhamii* [28]. General features of the sequenced trebouxiophyte organelle genomes, including those from *Coccomyxa*, are shown in Table 1.

**Table 1:** General features of available trebouxiophyte organelle genomes.

	Length (kb)	% GC	% NC	# Genes	# Introns (I/II)	Notable Features	GenBank Accession
**MtDNAs**							
*Coccomyxa*	65.4	53.2	52	59	1/4	High GC content. Shares repeats with ptDNA.	HQ874522
*Helicosporidium*	49.3	25.6	35	60	4/0	Fragmented *cox1* gene with trans-spliced group I intron.	GQ339576
*Pedinomonas minor*	25.1	22.2	43	22	0/1	Small, intronless genome. Reduced gene content. Fragmented *rrnS* gene.	NC_000892
*Prototheca wickerhamii*	55.3	25.8	34	61	5/0	Moderate size & coding content. High degree of synteny with *Helicosporidium* mtDNA.	NC_001613
**PtDNAs**							
*Chlorella vulgaris*	150.3	31.6	49	115	3/0	IR^−^. Moderate size & coding content.	NC_001865
*Coccomyxa*	175.7	50.7	56	115	1/0	IR^−^. Large genome with high GC content. Shares repeats with mtDNA.	HQ693844
*Helicosporidium*	37.4	26.9	5	54	1/0	IR^−^. Smallest & most reduced ptDNA from the Viridiplantae.	NC_008100
*Leptosira terrestris*	195.0	27.3	55	106	4/0	IR^−^. Largest available trebouxiophyte organelle DNA.	NC_009681
*Parachlorella kessleri*	123.9	30.0	36	112	1/0	IR^+^. Small, intron-poor genome.	NC_012978
*Pedinomonas minor*	98.3	38.4	29	105	0/0	IR^+^. Small, intronless genome.	FJ968740

Guanine and cytosine (GC); Noncoding DNA (NC), includes introns as well as intronic and unclassified ORFs; rRNA-, tRNA-, and protein-coding (Genes); Group I/group II introns (I/II); inverted repeat present (IR^+^) or absent (IR^−^).

With respective sizes of 65 and 175 kilobases (kb) and noncoding DNA contents of 52 and 56%, the *Coccomyxa* mitochondrial and plastid genomes are among the most inflated organelle DNAs observed from the Trebouxiophyceae, and are more akin to the prodigious organelles genomes often found within the Chlorophyceae and Ulvophyceae. Apart from the ptDNA of *L. terrestris*, which is 195 kb and 55% noncoding, most of the trebouxiophyte organelle genomes studied heretofore are relatively condensed (Table 1), as exemplified by the *P. minor* mtDNA, which is 25 kb and contains only 22 genes, and the *Helicosporidium* ptDNA, which is 37.4 kb and 95% coding, making it the most streamlined plastid genome observed from the Viridiplantae.

Annotation of the *Coccomyxa* organelle genomes revealed 59 (mtDNA) and 115 (ptDNA) putative genes, which are listed, along with the genes from the other sequenced trebouxiophyte organelle DNAs, in Supplementary Tables S1 and S2. Like other available trebouxiophyte organelle DNAs, save for the *P. minor* mtDNA, the *Coccomyxa* mitochondrial and plastid genomes use the standard genetic code. The mitochondrial gene content of *Coccomyxa* differs from those of *P. wickerhamii* and *Helicosporidium* in that it does not have *rpl6*, *rps11*, and *trnT*_ugu_. We identified intact copies of *rpl6* and *rps11* within the *Coccomyxa* nuclear genome sequence (v2) on scaffolds 12 and 36, respectively, but did not find an mtDNA-like copy of *trnT*_ugu_, leaving open the possibility that a nuclear-encoded cytosolic *trnT*_ugu_ is getting imported into the mitochondrion and compensating for the lost mitochondrial version of the gene. The ptDNA from *Coccomyxa* contains the same number of genes as that of *C. vulgaris*, which is about 3 to 10 more genes (mostly tRNAs) than those from other photosynthetic trebouxiophytes and around 50 more genes than the *Helicosporidium* plastid genome, which does not encode any photosynthetic proteins (Table 1; Supplementary Table S2).

Although replete with noncoding sequence, the *Coccomyxa* organelle genomes harbour few introns. We identified one group I intron in the ptDNA, found in *psbB*, and five introns in the mtDNA: a group I intron in *rrnL* and four group II introns, distributed among four tRNA-coding genes (*trnH*_gug_, *trnS*_gcu_, *trnS*_uga_, and *trnW*_cca_). Group II introns within tRNA-coding genes have been found in mtDNAs of other green algae, including those of *Chlorokybus atmophyticus*, *Chaetosphaeridium globosum*, and *C. vulgaris*. Three of tRNA group II introns in the *Coccomyxa* mtDNA (*trnH*_gug_, *trnS*_gcu_, *trnS*_uga_) are orthologous with those of *C. atmophyticus*. Low organelle intron contents are a common theme in trebouxiophytes and prasinophytes, whereas the organelle DNAs of chlorophyceans and ulvophytes are often intron dense.

Many of the intergenic regions in the *Coccomyxa* organelle genomes contain repetitive elements. Approximately 7% of both the mtDNA and ptDNA comprises repeats, most of which have forward (i.e., direct) or inverted (i.e., palindromic) orientations, are GC rich (60–70%), and are 20–250 nt in length (average 100 nt). Similar (even identical) repeats were found in both the mitochondrial and plastid compartments. In total, the *Coccomyxa* organelle genomes share ∼500 nt of repeat sequence with one another, distributed over approximately five different sites in the mtDNA and eleven in the ptDNA. To the best of our knowledge, this is one of only a few examples from all eukaryotes of a species harbouring the same repetitive element in both its mtDNA and ptDNA. GC-rich repeat elements have been identified in other organelle genomes, including the mtDNAs of the green algae *P. capuana* and *Chlorogonium elongatum* [3], [29]. The repeats within the *Coccomyxa* organelle DNAs, however, do not show any obvious sequence similarity to those from other organelle DNAs.

The plastid genome of *Coccomyxa*, like those of *C. vulgaris*, *L. terrestris*, and *Helicosporidium*, does not have a “quadripartite structure”, meaning that it lacks inverted repeats. The only sequenced trebouxiophyte ptDNAs that have inverted repeats are those of *Parachlorella kessleri* and *Pedinomonas minor* (Table 1), implying that loss of the inverted repeat has occurred multiple times throughout the evolution of trebouxiophytes [27].

### Organelle DNA nucleotide landscape

**Figure 2:**
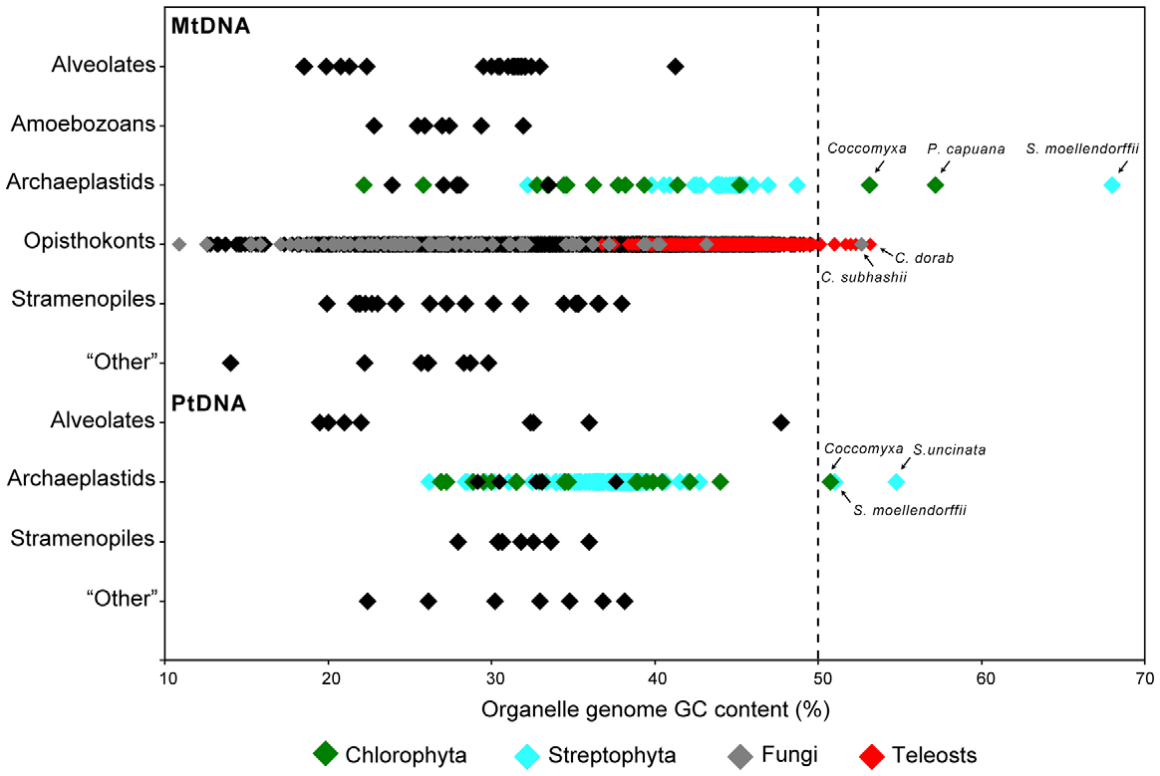
Guanine and cytosine compositions of completely sequenced mitochondrial and plastid genomes. Species with high organelle GC contents are labeled (see Table 2 for full genus names). Organelle genome sequences were downloaded from the GenBank Reference Sequence collection on 1 March 2011.

The *Coccomyxa* organelle genomes have overall GC contents of 53.2% (mtDNA) and 50.7% (ptDNA). Although only modestly biased in G and C, the nucleotide compositions of these two genomes are made more impressive by the AT bias found in nearly all other available mtDNA and ptDNA sequences (Figure 2). Indeed, *Coccomyxa* belongs to a small but eclectic list of species that are known to have organelle DNAs that exceed 50% GC (Table 2).

**Table 2:** General features of available GC-rich organelle genomes.

	Lineage	GC total	GC1	GC2	GC3	GC coding/intergenic	Notable Features	GenBank Accession
**MtDNAs**								
*Candida subhashii*	Yeast	52.7	53.2	38.7	66.4	52.3/54.4	Linear genome with inverted repeat telomeres. Pathogenic species.	NC_014337
*Chirocentrus dorab*	Teleost	53.2	58.7	47.7	53.7	53.6/39.3	Compact, intronless genome.	NC_006913
*Coccomyxa*	Green alga	53.2	51.4	40.8	59.8	51.1/55.7	GC-rich repeat elements. Similar repeats in both mtDNA and ptDNA.	HQ874522
*Polytomella capuana*	Green alga	57.2	52.2	41.3	76.0	56.4/61.0	Highly reduced, linear genome with inverted repeat telomeres. Nonphotosynthetic species.	NC_010357
*Selaginella moellendorffii*	Land plant	68.1	64.2	60.2	61.5	63.5/68.9	Extreme C-to-U RNA editing. Complex network of recombinogenic mtDNA molecules.	GQ246802-8 JF338143-7
**PtDNAs**								
*Coccomyxa*	Green alga	50.7	56.1	43.6	50.1	50.3/51.0	GC-rich repeat elements. Similar repeats in both mtDNA and ptDNA.	NC_015084
*Selaginella moellendorffii*	Land plant	51.0	55.9	50.8	44.8	51.4/49.9	Extreme C-to-U RNA editing	NC_013086
*Selaginella uncinata*	Land plant	54.8	58.8	54.7	49.3	54.8/54.9	Extreme C-to-U RNA editing	AB197035

Percentage of guanine and cytosine (GC) of entire genome (total), first-, second-, and third-position codon sites (GC1, GC2, and GC3), rRNA-, tRNA-, and protein-coding regions (coding), and intergenic regions (intergenic).

The individual strands of the *Coccomyxa* organelle genomes contain almost equal proportions of G versus C (the GC skew of the mtDNA is 0.015 and that of the ptDNA is 0.008), a trait that is shared by other GC-rich organelle DNAs, except for the mtDNA of the wolf herring *Chirocentrus dorab*, which contains an abundance of cytosine on the main sense strand. Analyses of *Coccomyxa* EST sequence data revealed no evidence of post-transcriptional editing in either the mitochondrial or plastid compartments (∼10 organelle transcripts were explored), contrasting the massive levels of C-to-U editing found in the GC-biased mtDNA and ptDNA of the spikemoss *Selaginella moellendorffii* [7]–[9]. *Selaginella* organelle genomes are currently the only examples where GC richness is allied with post-transcriptional editing (Table 2).

The GC composition differs among the various regions of the *Coccomyxa* organelle genomes (Table 2). For the mtDNA, the GC contents of intergenic regions (55.7%) and third codon positions (59.8%) are higher than those of the more functionally constrained regions, such as first and second codon positions (51.4 and 40.8%) and sites coding for structural RNAs (52.5%). In the plastid genome the GC content at first codon positions (56.1%) exceeds that of intergenic (51%) and third codon positions (50.1%), and is lowest at second codon positions (43.6%). An inflated GC content at first codon positions relative to the other genomic regions is a general trend among plastid genomes (reflecting, in part, an amino acid composition rich in leucine) and is not unique to *Coccomyxa* (see [7] for a compilation of the codon position GC contents for various completely sequenced ptDNAs). For both the mtDNA and ptDNA, the highest levels of G and C are observed for the repetitive elements, most of which are between 60–75% GC.

The deduced amino acid sequences of the *Coccomyxa* mtDNA and ptDNA protein-coding genes, although slightly enriched in alanine and glycine, are not markedly different from their counterparts in AT-rich trebouxiophyte organelle DNAs (Supplementary Tables S3 and S4). It is therefore unlikely that the GC contents of the *Coccomyxa* organelle genomes are driven by selection for a particular amino acid composition. Moreover, the tRNA repertoire and anticodon suite encoded within the organelle DNAs of *Coccomyxa* are almost identical to those from other AT-rich trebouxiophyte organelle genomes (Supplementary Tables S1 and S2). Adaptation for DNA thermo stability (resulting from the extra hydrogen bond in G•C pairs) can also be ruled out as *Coccomyxa* sp. 169 originates from Marble Point Antarctica [30]. So then what is the nature of the forces biasing the *Coccomyxa* organelle DNAs in G and C? When addressing this question, it may help to reflect on why most organelle genomes are enriched in A and T.

The forces shaping organelle genome nucleotide landscape are poorly understood and probably differ both among and within lineages. Some have invoked selection for translational efficiency to explain the high levels of A and T in organelle genomes (reviewed in [31]), whereas others contend that it stems from AT mutation pressure coupled with inefficient mitochondrial and plastid DNA repair processes [31]–[33]. Indeed, mitochondrial and plastid genomes, because they are housed in energy-producing organelles, are often exposed to reactive oxygen species (ROS), which, by causing the deamination of cytosine to uracil and the oxidative conversion of guanine to 8-oxo-guanine, promote C∶G→T∶A transitions and C∶G→A∶T transversions [31]. That being said, the plastid genomes from nonphotosynthetic species, like *Helicosporidium* and *Plasmodium falciparum*, which are not currently exposed to high levels of ROS, are also AT rich, suggesting that there are a diversity of forces biasing organelle genomes towards A and T. Whatever these forces may be, *Coccomyxa* appears to be buffering or counteracting them. The following observations give insight into how it may be achieving this.

Within the *Coccomyxa* mtDNA and ptDNA there are, with some exceptions, an excess of guanines and cytosines at silent sites relative to the more functionally constrained positions (Table 2). This distribution of G and C is best explained by the negative selection principle of the neutral theory of molecular evolution [34]. In other words, nonadaptive forces may be driving the nucleotide composition towards G and C. The two neutral processes that are thought to influence nucleotide landscape are biased mutation pressure, which, as discussed above, seems to be skewed towards A and T in mitochondria and plastids, and biased gene conversion, which favours G and C in nuclear genomes [35], [36] but appears to be AT-biased in the sole organelle system in which it has been studied (the ptDNA of tobacco) [37]. Although speculative, one possibility is that the inflated GC content of the *Coccomyxa* organelle DNA repeat elements — sequences that presumably undergo high levels of recombination and therefore experience a lot of gene conversion [38] — may be a sign of a GC-biased conversion process within the mitochondrion and plastid of this species; however, this would contrast the AT biased conversion process found in the tobacco plastid genome [37]. Also, the presence of identical GC-rich repeats in both the mtDNA and ptDNA of *Coccomyxa* implies that there is a mobile nature to at least some of these elements, meaning that they may be able to propagate their GC richness by spreading their sequence throughout the various noncoding regions.

Another possibility is that a mutation conferring a GC-biased mutational pattern has been fixed in *Coccomyxa*. Given that both the mtDNA and ptDNA are GC rich, one would expect that this new mutation bias be derived from a system that affected both organelles. The mitochondrial and plastid compartments share comparatively few proteins with one another, but nucleus-encoded dual-targeted organelle proteins have been characterized in a number of lineages, and are the rule rather than the exception in some systems [39], [40]. The similar GC bias of the mtDNA and ptDNA may imply that cell-wide features, like life history characteristics, environment, and/or metabolic processes, have influenced the mitochondrial and plastid nucleotide compositions. In this context, it is noteworthy that the overall GC content of *Coccomyxa* nuclear genome (∼53%) is comparable to that of the organelle DNAs.

Organelle DNA sequence data from other *Coccomyxa* species are scarce, but we were able to find in GenBank partial *rbcL* ptDNA sequences from 49 different *Coccomyxa* strains. Forty-seven of these strains have relatively GC-poor *rbcL* sequences (average = 40% GC), suggesting that their plastid genomes are AT rich. However, the *rbcL* GC contents from two strains — *Coccomyxa chodatii* (49%) and *Coccomyxa rayssiae* (47.3%) — are similar to that of *Coccomyxa* sp. C-169 (49.3%), the focus of this study. All three of these taxa are non-lichenized, free living species, whereas the other 47 species, which had typical AT-rich *rbcL* GC contents, are symbionts.

### Phylogenetic analyses

Sparse taxon sampling and a lack of DNA sequence data have made it difficult to resolve the phylogenetic relationships among the Ulvophyceae, Trebouxiophyceae, and Chlorophyceae. The completion of the *Coccomyxa* mitochondrial and plastid genomes allows for a more thorough phylogenetic evaluation of these groups. Using two amino acid sequence datasets — one with 29 mtDNA-encoded proteins and another containing 67 ptDNA-encoded proteins — we performed Maximum Likelihood (LG+Γ model) and Bayesian phylogenetic (CAT+GTR+Γ model) analyses on *Coccomyxa* and various other green algae for which complete or almost complete organelle DNA sequences are available. *Helicosporidium* was not included in the phylogenetic analyses because it lacked several important organelle genes for phylogenetic reconstruction; when we included *Helicosporidium* (which groups with species from the Chlorellaceae) in our analyses it did not alter the tree topologies discussed below.

**Figure 3:**
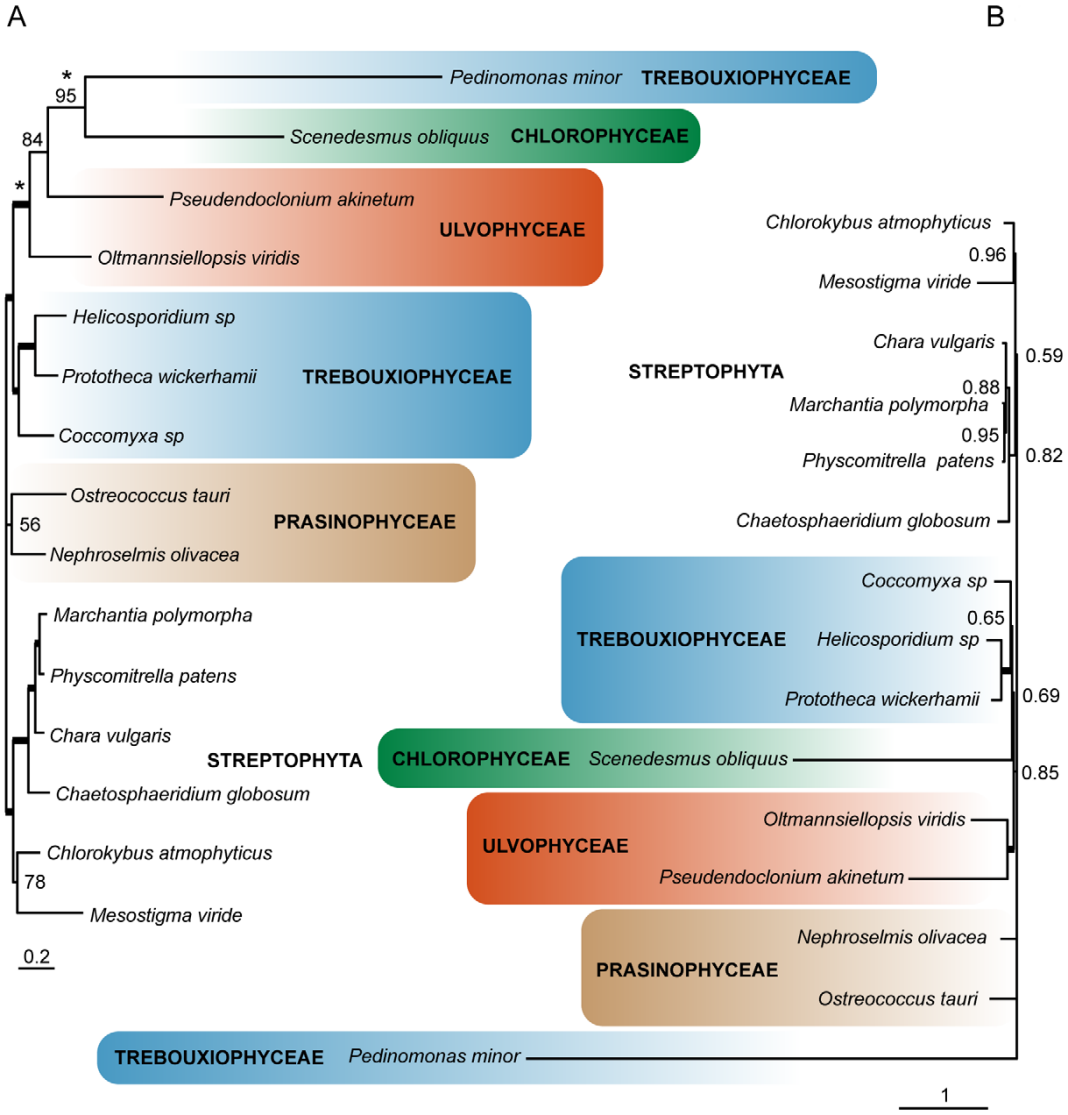
Maximum-likelihood (A) and Bayesian (B) trees inferred from the amino acid sequence of 29 mtDNA-encoded proteins. Phylogenetic tree of green algae, with 6 streptophyte species used as outgroup. Thick branches represent maximal statistical support (100% bootstrap support in A; 1.0 Bayesian posterior probabilities in B). When not maximal, values are indicated. The scale bar represents the estimated number of amino acid substitutions per site.

**Figure 4:**
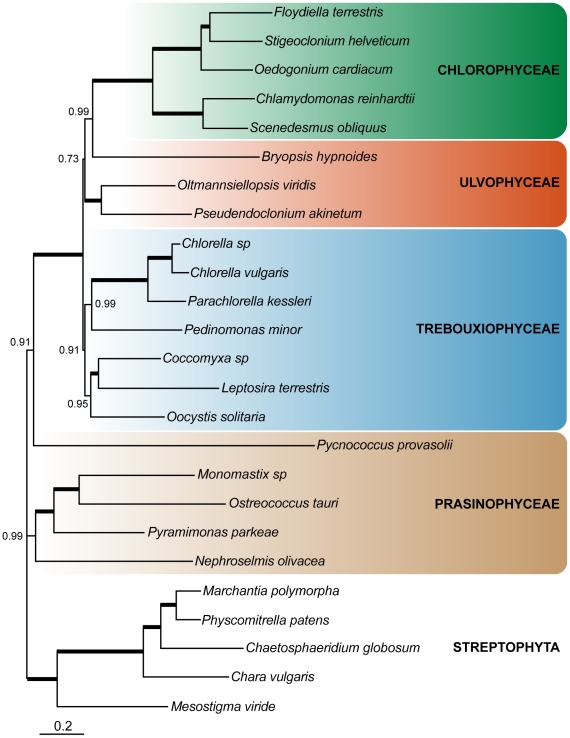
Bayesian tree inferred from the amino acid sequence of 67 ptDNA-encoded proteins. Phylogenetic tree of green algae, with 5 streptophyte species used as outgroup. Thick branches represent maximal statistical support (1.0 Bayesian posterior probabilities). When not maximal, values are indicated. The scale bar represents the estimated number of amino acid substitutions per site.

Both the mitochondrial and plastid phylogenies (Figures 3 and 4, respectively) consistently grouped *Coccomyxa* with other trebouxiophytes. On the mitochondrial trees, *Coccomyxa* was sister to a clade containing *Helicosporidium* and *P. wickerhamii*, but the taxon sampling remains relatively poor, and elevated rates of evolution for *P. minor* and *Scenedesmus obliquus* prevented resolution of the UTC classes. The grouping of *P. minor* with *Scenedesmus obliquus* received high bootstrap support (95%) in the ML tree (Figure 3A), as it did in previous mtDNA-based green algal phylogenies [19], [20], but given that both of these species show high rates of mtDNA substitution, we argue that this arrangement is caused by long-branch attraction (LBA), which also appears to be attracting the relatively fast evolving ulvophyte *P. akinetum*, resulting in a paraphyletic Ulvophyceae (Figure 3A). Our concerns regarding LBA were confirmed by Bayesian inference with the site-heterogeneous CAT model [combined with a general time reversible (GTR) exchange rate matrix and gamma correction], which has been shown to handle saturated positions more efficiently than site-homogeneous models such as the LG matrix [41]. In the Bayesian tree (Figure 3B), a monophyletic Ulvophyceae clade was recovered, with maximal statistical support, and the position of *P. minor* and *S. obliquus* were unresolved.

The plastid dataset contained both a larger diversity of chlorophyte species and more than twice the number of genes than the mitochondrial dataset. Figure 4 corresponds to the Bayesian tree inferred from the site-heterogeneous CAT+GTR+Γ model. On this tree the trebouxiophytes formed a monophyletic group comprising two subclades: one containing *P. minor* and members of the Chlorellaceae and another harbouring *Coccomyxa*, *O. solitaria*, and *L. terrestris*. Previous plastid multi-gene phylogenies positioned *L. terrestris* on a long branch next to the Chlorophyceae [18], [42], but our analyses place it with the trebouxiophytes and indeed sister to *Coccomyxa* with high support, which is consistent with its traditional classification [43]. The plastid tree also recovered a weakly supported sister relationship between the Ulvophyceae and Chlorophyceae, with the Trebouxiophyceae branching at the base of the chlorophyte crown. Although the relationships among the UTC groups were not burdened by dramatically long branches, it is noticeable that the Chlorophyceae in general and the ulvophyte *Bryopsis hypnoides* evolve faster than the rest of the UTC members. In an attempt to test for an undetected misleading effect resulting from this heterogeneous rate of sequence evolution that would lower the support for the overall UTC branching pattern, we reconstructed a tree where six fast-evolving lineages were discarded: *P. provasolii*, *B. hypnoides* and all Chlorophyceae except *S. obliquus* (Supplementary Figure S1). Using this reduced dataset, the statistical support for the Ulvophyceae and Chlorophyceae grouping increased significantly (0.73 PP to 0.99 PP); the monophyly of Trebouxiophyceae, however, remained poor. This branching order is consistent with previous phylogenetic analyses using 18S rRNA [17], [44] and plastid genome architectural features [18] and reinforces the belief that the ancestor of the UTC clade had a phycoplast and a non-persistent mitotic spindle [45] — features that were presumably lost in the Ulvophyceae. An early diverging Trebouxiophyceae also implies that a counter-clockwise flagellar orientation was an ancestral characteristic of the UTC, which evolved to directly opposed and clockwise orientations within the Chlorophyceae. This hypothesis is consistent with the counter clockwise flagellar root system in the prasinophyte class Chlorodendrophyceae [45].

## Materials and Methods

### Strain information

*Coccomyxa* sp. C-169 (formerly referred to as *Chlorella* sp. C-169 and then redefined after phylogenetic analysis of the draft nuclear genome sequence [DOE JGI, unpublished data]) was maintained at the culture collection of the Institute of Molecular and Cellular Biosciences (IAM), University of Tokyo, until 2007. It is now available from the Microbial Culture Collection at the National Institute for Environmental Studies (NIES) under strain number 2166. *Coccomyxa* C-169/NIES-2166 originates from Marble Point Antarctica.

### Assembly and annotation of the Coccomyxa organelle genomes

The *Coccomyxa* organelle genomes were sequenced as a part of the DOE JGI *Coccomyxa* genome project using whole-genome shotgun sequencing on a Sanger platform. Reads were data-mined from the National Center for Biotechnology Information (NCBI) *Coccomyxa* Trace Archive using the following sequences as BlastN (v2.2.25+) queries: the *Helicosporidium* and *P. wickerhamii* mitochondrial genomes and the *C. vulgaris* ptDNA. BlastN parameters were as follows: an expectation value (E-value) of 0.0001; a word size of 11; match and mismatch scores of 2 and −3, respectively; and gap-cost values of 5 (existence) and 2 (extension). Trace files corresponding to Blast hits were assembled with CodonCode Aligner v3.7.1.1 (CodonCode Corporation, Dedham, MA, USA). Gaps in the assemblies were filled by blasting the generated *Coccomyxa* mtDNA and ptDNA contigs against the *Coccomyxa* Trace Archive and then using the hits to extend the contigs. The entire *Coccomyxa* organelle genome sequences were blasted against the *Coccomyxa* draft nuclear genome sequence (v2) to verify that they were not generated from either nuclear mitochondrial DNAs or nuclear plastid DNAs. These analyses indicate that the mitochondrial and plastid genome assemblies were generated from organelle DNA and are consistent with the view that *Coccomyxa* harbours very little organelle-like sequences in its nuclear genome [46].

Introns and repeat elements were respectively detected with RNAweasel [47] and REPuter [48]. Additional scans for repeats were performed by building custom BLAST databanks of the *Coccomyxa* mtDNA and ptDNA sequences and then blasting (BlastN v2.2.25+) these with specific regions from the mitochondrial and plastid genomes. *Coccomyxa* EST and nuclear genome sequence data (v2) were downloaded from the DOE JGI *Coccomyxa* genome portal.

### Phylogenetic analyses

The phylogenetic datasets included the concatenated alignments of 29 mtDNA-encoded genes (5,344 amino acids) from 15 taxa (mitochondrial dataset) and 67 ptDNA-encoded genes (1,1218 amino acids) from 25 taxa (plastid dataset); see Supplementary Table S5 for a list of species and genes that were employed. Sequences were aligned using the L-INS-I method of the MAFFT package [49]. Poorly aligned positions were removed with Gblocks [50] using the following settings: no gaps allowed, minimum number of sequences for conserved and flank positions equal to 50% of the number of taxa plus one, a maximum number of contiguous non-conserved positions of 10, and a minimum block length of 8. *Chlamydomonas reinhardtii* was discarded from the mitochondrial dataset because it showed high rates of mtDNA evolution and has a gene-poor mitochondrial genome — a more slowly evolving representative for the Chlorophyceae was available (i.e., *S. obliquus*). The *Helicosporidium* ptDNA was not included in the plastid dataset because it is highly reduced and thus lacks several genes that were important for phylogenetic reconstruction. The final gene datasets were based on the presence of at least 70% of the species that were to be included in the final concatenations (Supplementary Table S5).

Maximum Likelihood (ML) phylogenetic analyses were run with RAxML 7.2.8 [51] in combination with the rapid hill-climbing algorithm and the site-homogeneous LG+Γ+F model of evolution (−m PROTGAMMALGF, 4 discrete rate categories). The best-scoring ML tree was based on multiple searches of 20 randomized stepwise addition parsimony starting trees. Statistical support was evaluated with non-parametric bootstrapping using 100 replicates. Bayesian phylogenetic analyses were performed with PhyloBayes 3.2f [52] using the site-heterogeneous mixture CAT model with a general time reversible (GTR) exchange rate matrix and gamma correction (4 categories). Two independent Markov chains with a total length of 10,000 cycles were performed, discarding the first 2000 points as burnin, and calculating the posterior consensus on the remaining trees. Convergence between the two chains was ascertained by examining the difference in frequency for all of their bipartitions (<0.05 in all analyses).

For the fast-evolving species removal analysis on the plastid dataset, root-to-tip distances were calculated with the “ape” and “geiger” packages available in R, with a root position defined on the branch leading to the monophyletic clade of *Marchantia polymorpha* and *Physcomitrella patens* (within the outgroup Streptophyta). Taxa were sorted according to their distance to the root, and the fastest evolving taxa removed (*Pycnococcus provasolii*, *Bryopsis hypnoides*, and all of the Chlorophyceae except the relatively slowly evolving *S. obliquus*).

## Supporting Information

Figure S1 Bayesian phylogenetic tree of the ptDNA data with fast-evolving species removed
(PDF)

Table S1 Gene contents of available trebouxiophyte mitochondrial genomes
(PDF)

Table S2 Gene contents of available trebouxiophyte plastid genomes
(PDF)

Table S3 Amino acid composition of proteins encoded in complete mitochondrial genome sequences from trebouxiophytes
(PDF)

Table S4 Amino acid composition of proteins encoded in complete plastid genome sequences from trebouxiophytes
(PDF)

Table S5 Gene and species lists for the mitochondrial and chloroplast datasets
(XLSX)
