# Supplementary figures and images for: The GC-Rich Mitochondrial and Plastid Genomes of the Green Alga Coccomyxa Give Insight into the Evolution of Organelle DNA Nucleotide Landscape
Source: PLoS One. 2011 Aug 26;6(8):e23624. doi: 10.1371/journal.pone.0023624 (PMC3162594; doi:10.1371/journal.pone.0023624)

Figure S1

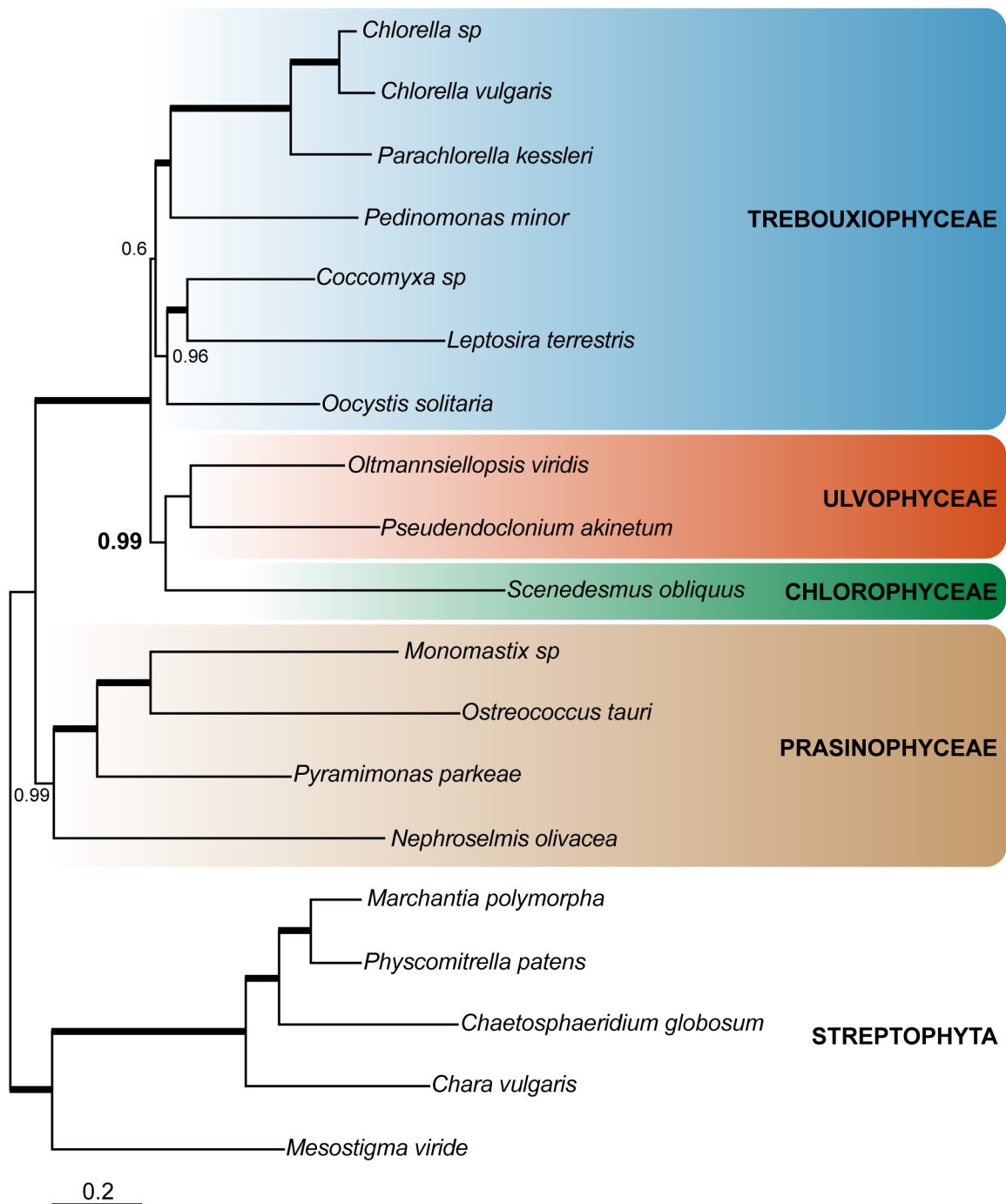

Supplement: Figure S1 — Bayesian phylogenetic tree of the ptDNA data with fast-evolving species removed (PDF) [file pone.0023624.s001.pdf]
