# Supplementary material for: The GC-Rich Mitochondrial and Plastid Genomes of the Green Alga Coccomyxa Give Insight into the Evolution of Organelle DNA Nucleotide Landscape
Source: PLoS One. 2011 Aug 26;6(8):e23624. doi: 10.1371/journal.pone.0023624 (PMC3162594; doi:10.1371/journal.pone.0023624)
Supplement: Table S1 — Gene contents of available trebouxiophyte mitochondrial genomes (PDF) [file pone.0023624.s002.pdf]

Table S1. Gene contents of available trebouxiophyte mitochondrial genomes

Coccomyxa	***Helicosporidium***	***Prototheca wickerhamii***	***Pedinomonas minor***
atp1	*atp1*	*atp1*	—
atp4	*atp4*	*atp4*	—
atp6	*atp6*	*atp6*	*atp6*
atp8	*atp8*	*atp8*	*atp8*
atp9	*atp9*	*atp9*	—
cob	*cob*	*cob*	*cob*
cox1	*cox1*	*cox1*	*cox1*
cox2	*cox2*	*cox2*	—
cox3	*cox3*	*cox3*	—
nad1	*nad1*	*nad1*	*nad1*
nad2	*nad2*	*nad2*	*nad2*
nad3	*nad3*	*nad3*	*nad3*
nad4	*nad4*	*nad4*	*nad4*
nad4L	*nad4L*	*nad4L*	*nad4L*
nad5	*nad5*	*nad5*	*nad5*
nad6	*nad6*	*nad6*	*nad6*
nad7	*nad7*	*nad7*	—
nad9	*nad9*	*nad9*	—
rrn5	*rrn5*	*rrn5*	—
rrnL	*rrnL*	*rrnL*	*rrnL*
rrnS	*rrnS*	*rrnS*	*rrnS* (fragmented)
rpl5	*rpl5*	*rpl5*	—
—	*rpl6*	*rpl6*	—
rpl16	*rpl16*	*rpl16*	—
rps2	*rps2*	*rps2*	—
rps3	*rps3*	*rps3*	—
rps4	*rps4*	*rps4*	—
rps7	*rps7*	*rps7*	—
rps10	*rps10*	*rps10*	—
—	*rps11*	*rps11*	—
rps12	*rps12*	*rps12*	—
rps13	*rps13*	*rps13*	—
rps14	*rps14*	*rps14*	—
rps19	*rps19*	*rps19*	—
tatC	*tatC*	*tatC*	—
trnA(ugc)	*trnA(ugc)*	*trnA(ugc)*	—
trnC(gca)	*trnC(gca)*	*trnC(gca)*	*trnC(gca)*
trnD(guc)	*trnD(guc)*	*trnD(guc)*	—
trnE(uuc)	*trnE(uuc)*	*trnE(uuc)*	*trnE(uuc)*
trnF(gaa)	*trnF(gaa)*	*trnF(gaa)*	*trnF(gaa)*

Coccomyxa	***Helicosporidium***	***Prototheca wickerhamii***	***Pedinomonas minor***
trnG(gcc)	—	*trnG(gcc)*	—
trnG(ucc)	*trnG(ucc)*	*trnG(ucc)*	—
trnH(gug)	*trnH(gug)*	*trnH(gug)*	*trnH(gug)*
trnI(gau)	*trnI(gau)*	*trnI(gau)*	—
trnI(cau)	*trnI(cau)*	*trnI(cau)*	—
trnK(uuu)	*trnK(uuu)*	*trnK(uuu)*	—
trnL(caa)	*trnL(uaa)*	*trnL(uaa)*	*trnL(caa)*
trnL(uaa)	*trnL(uag)*	*trnL(uag)*	—
trnL(uag)	—	—	—
trnMe(cau)	*trnMe(cau)*	*trnMe(cau)*	—
trnMf(cau)	*trnMf(cau)*	*trnMf(cau)*	—
trnN(guu)	*trnN(guu)*	*trnN(guu)*	—
trnP(ugg)	*trnP(ugg)*	*trnP(ugg)*	—
trnQ(uug)	*trnQ(uug)*	*trnQ(uug)*	*trnQ(uug)*
trnR(ucu)	*trnR(ucu)*	*trnR(ucu)*	—
trnR(acg)	*trnR(acg)*	*trnR(acg)*	—
trnS(gcu)	*trnS(gcu)*	*trnS(gcu)*	—
trnS(uga)	*trnS(uga)*	*trnS(uga)*	—
—	*trnT(ugu)*	*trnT(ugu)*	—
trnV(uac)	*trnV(uac)*	*trnV(uac)*	—
trnW(cca)	*trnW(cca)*	*trnW(cca)*	*trnW(uca)*
trnY(gua)	*trnY(gua)*	*trnY(gua)*	*trnY1(gua)*
—	—	—	*trnY2(gua)*
