# Supplementary material for: The GC-Rich Mitochondrial and Plastid Genomes of the Green Alga Coccomyxa Give Insight into the Evolution of Organelle DNA Nucleotide Landscape
Source: PLoS One. 2011 Aug 26;6(8):e23624. doi: 10.1371/journal.pone.0023624 (PMC3162594; doi:10.1371/journal.pone.0023624)
Supplement: Table S2 — Gene contents of available trebouxiophyte plastid genomes (PDF) [file pone.0023624.s003.pdf]

Table S2. Gene contents of available trebouxiophyte plastid genomes

Coccomyxa	***Chlorella vulgaris***	***Helicosporidium***	***Parachlorella kessleri***	***Leptosira terrestris***	***Pedinomonas minor***
accD	*accD*	*accD*	*accD*	*accD*	*accD*
atpA	*atpA*	—	*atpA*	*atpA*	*atpA*
atpB	*atpB*	—	*atpB*	*atpB*	*atpB*
atpE	*atpE*	—	*atpE*	*atpE*	*atpE*
atpF	*atpF*	—	*atpF*	*atpF*	*atpF*
atpH	*atpH*	—	*atpH*	*atpH*	*atpH*
atpI	*atpI*	—	*atpI*	*atpI*	*atpI*
ccsA	*ccsA*	—	*ccsA*	—	*ccsA*
cemA	*cemA*	—	*cemA*	*cemA*	*cemA*
chlB	*chlB*	—	*chlB*	*chlB*	—
chlI	*chlI*	—	*chlI*	—	*chlI*
chlL	*chlL*	—	*chlL*	*chlL*	—
chlN	*chlN*	—	*chlN*	*chlN*	—
clpP	*clpP*	—	*clpP*	*clpP*	*clpP*
cysA	*cysA*	—	*cysA*	*cysA*	*cysA*
cysT	*cysT*	*cysT*	*cysT*	*cysT*	*cysT*
ftsH	*ftsH*	*ftsH*	*ftsH*	*ftsH*	*ftsH*
infA	*infA*	—	*infA*	*infA*	—
minD	*minD*	—	*minD*	*minD*	*minD*
petA	*petA*	—	*petA*	*petA*	*petA*
petB	*petB*	—	*petB*	*petB*	*petB*
petD	*petD*	—	*petD*	*petD*	*petD*
petG	*petG*	—	*petG*	*petG*	*petG*
petL	*petL*	—	*petL*	*petL*	*petL*
psaA	*psaA*	—	*psaA*	*psaA*	*psaA*

Coccomyxa	***Chlorella vulgaris***	***Helicosporidium***	***Parachlorella kessleri***	***Leptosira terrestris***	***Pedinomonas minor***
psaB	*psaB*	—	*psaB*	*psaB*	*psaB*
psaC	*psaC*	—	*psaC*	*psaC*	*psaC*
psaI	*psaI*	—	*psaI*	*psaI*	*psaI*
psaJ	*psaJ*	—	*psaJ*	*psaJ*	*psaJ*
psaM	*psaM*	—	*psaM*	*psaM*	*psaM*
psbA	*psbA*	—	*psbA*	*psbA*	*psbA*
psbB	*psbB*	—	*psbB*	*psbB*	*psbB*
psbC	*psbC*	—	*psbC*	*psbC*	*psbC*
psbD	*psbD*	—	*psbD*	*psbD*	*psbD*
psbE	*psbE*	—	*psbE*	*psbE*	*psbE*
psbF	*psbF*	—	*psbF*	*psbF*	*psbF*
psbH	*psbH*	—	*psbH*	*psbH*	*psbH*
psbI	*psbI*	—	*psbI*	*psbI*	*psbI*
psbJ	*psbJ*	—	*psbJ*	*psbJ*	*psbJ*
psbK	*psbK*	—	*psbK*	*psbK*	*psbK*
psbL	*psbL*	—	*psbL*	*psbL*	*psbL*
psbM	*psbM*	—	*psbM*	*psbM*	*psbM*
psbN	*psbN*	—	*psbN*	*psbN*	*psbN*
psbT	*psbT*	—	*psbT*	*psbT*	*psbT*
psbZ	*psbZ*	—	*psbZ*	*psbZ*	*psbZ*
rbcL	*rbcL*	—	*rbcL*	*rbcL*	*rbcL*
rpl12	*rpl12*	*rpl12*	*rpl12*	*rpl12*	*rpl12*
rpl14	*rpl14*	*rpl14*	*rpl14*	*rpl14*	*rpl14*
rpl16	*rpl16*	*rpl16*	*rpl16*	*rpl16*	*rpl16*
rpl19	*rpl19*	—	*rpl19*	*rpl19*	*rpl19*
rpl2	*rpl2*	*rpl2*	*rpl2*	*rpl2*	*rpl2*

Coccomyxa	***Chlorella vulgaris***	***Helicosporidium***	***Parachlorella kessleri***	***Leptosira terrestris***	***Pedinomonas minor***
rpl20	*rpl20*	*rpl20*	*rpl20*	*rpl20*	*rpl20*
rpl23	*rpl23*	—	*rpl23*	*rpl23*	*rpl23*
rpl32	*rpl32*	*rpl32*	*rpl32*	*rpl32*	*rpl32*
rpl36	*rpl36*	*rpl36*	*rpl36*	*rpl36*	*rpl36*
rpl5	*rpl5*	*rpl5*	*rpl5*	*rpl5*	*rpl5*
rpoA	*rpoA*	*rpoA*	*rpoA*	*rpoA*	*rpoA*
rpoB	*rpoB*	*rpoB*	*rpoB*	*rpoBa*	*rpoB*
—	—	—	—	*rpoBb*	—
rpoC1	*rpoC1*	*rpoC1*	*rpoC1*	*rpoC1*	*rpoC1*
rpoC2	*rpoC2*	*rpoC2*	*rpoC2*	*rpoC2*	*rpoC2*
rps11	*rps11*	*rps11*	*rps11*	*rps11*	*rps11*
rps12	*rps12*	*rps12*	*rps12*	*rps12*	*rps12*
rps14	*rps14*	*rps14*	*rps14*	*rps14*	*rps14*
rps18	*rps18*	—	*rps18*	*rps18*	*rps18*
rps19	*rps19*	*rps19*	*rps19*	*rps19*	*rps19*
rps2	*rps2*	—	*rps2*	*rps2*	*rps2*
rps3	*rps3*	*rps3*	*rps3*	*rps3*	*rps3*
rps4	*rps4*	*rps4*	*rps4*	*rps4*	*rps4*
rps7	*rps7*	*rps7*	*rps7*	*rps7*	*rps7*
rps8	*rps8*	*rps8*	*rps8*	*rps8*	*rps8*
rps9	*rps9*	—	*rps9*	*rps9*	*rps9*
rrn5	*rrn5*	*rrn5*	*rrn5*	*rrn5*	*rrn5*
rrnL	*rrnL*	*rrnL*	*rrnL*	*rrnL*	*rrnL*
rrnS	*rrnS*	*rrnS*	*rrnS*	*rrnS*	*rrnS*
tilS (fragment 1)	*tilS*	*tilS*	*tilS*	*tilS*	—
tilS (fragment 2)	—	—	—	—	—

Coccomyxa	***Chlorella vulgaris***	***Helicosporidium***	***Parachlorella kessleri***	***Leptosira terrestris***	***Pedinomonas minor***
trnA(ugc)	*trnA(ugc)*	*trnA(ugc)*	*trnA(ugc)*	*trnA(ugc)*	*trnA(ugc)*
trnC(gca)	*trnC(gca)*	*trnC(gca)*	*trnC(gca)*	*trnC(gca)*	*trnC(gca)*
trnD(guc)	*trnD(guc)*	*trnD(guc)*	*trnD(guc)*	*trnD(guc)*	*trnD(guc)*
trnE(uuc)	*trnE(uuc)*	*trnE(uuc)*	*trnE(uuc)*	*trnE(uuc)*	*trnE(uuc)*
trnF(gaa)	*trnF(gaa)*	*trnF(gaa)*	*trnF(gaa)*	*trnF(gaa)*	*trnF(gaa)*
trnG(ucc)	*trnG(ucc)*	*trnG(ucc)*	*trnG(gcc)*	*trnG(gcc)*	*trnG(gcc)*
trnG(gcc)	*trnG(gcc)*	—	*trnG(ucc)*	*trnG(ucc)*	*trnG(ucc)*
—	*trnG(gcc)*	—	—	—	—
trnH(gug)	*trnH(gug)*	*trnH(gug)*	*trnH(gug)*	*trnH(gug)*	*trnH(gug)*
trnI(gau)	*trnI(gau)*	*trnI(gau)*	*trnI(gau)*	*trnI(gau)*	*trnI(gau)*
trnI(cau)	*trnI(cau)*	*trnI(cau)*	*trnI(cau)*	*trnI(cau)*	—
trnK(uuu)	*trnK(uuu)*	*trnK(uuu)*	*trnK(uuu)*	*trnK(uuu)*	*trnK(uuu)*
trnL(caa)	*trnL(caa)*	*trnL(uaa)*	*trnL(caa)*	*trnL(caa)*	—
trnL(gag)	*trnL(gag)*	—	*trnL(gag)*	—	—
trnL(uaa)	—	—	*trnL(uaa)*	*trnL(uaa)*	*trnL(uaa)*
trnL(uag)	*trnL(uag)*	*trnL(uag)*	*trnL(uag)*	*trnL(uag)*	*trnL(uag)*
trnMe(cau)	*trnMe(cau)*	*trnMe(cau)*	*trnMe(cau)*	*trnMe(cau)*	*trnMe(cau)*
trnMf(cau)	*trnMf(cau)*	*trnMf(cau)*	*trnMf(cau)*	*trnMf(cau)*	*trnMf(cau)*
trnN(guu)	*trnN(guu)*	*trnN(guu)*	*trnN(guu)*	*trnN(guu)*	*trnN(guu)*
trnP(ugg)	*trnP(ugg)*	*trnP(ugg)*	*trnP(ugg)*	*trnP(ugg)*	*trnP(ugg)*
trnQ(uug)	*trnQ(uug)*	*trnQ(uug)*	*trnQ(uug)*	*trnQ(uug)*	*trnQ(uug)*
trnR(acg)	*trnR(acg)*	*trnR(acg)*	*trnR(acg)*	*trnR(acg)*	*trnR(acg)*
trnR(ucu)	*trnR(ucu)*	*trnR(ucu)*	*trnR(ucu)*	*trnR(ucu)*	*trnR(ucu)*
trnR(ccg)	*trnR(ccg)*	—	—	*trnR(ccg)*	*trnR(ccg)*
trnR(ccu)	—	—	—	—	—
trnS(gcu)	*trnS(gcu)*	—	*trnS(gcu)*	*trnS(gcu)*	*trnS(gcu)*

Coccomyxa	*Chlorella vulgaris*	*Helicosporidium*	*Parachlorella kessleri*	*Leptosira terrestris*	*Pedinomonas minor*
trnS(gga)	*trnS(gga)*	*trnS(uga)*	*trnS(gga)*	—	*trnS(gga)*
trnS(uga)	*trnS(uga)*	*trnS(gcu)*	*trnS(uga)*	*trnS(uga)*	*trnS(uga)*
trnT(ugu)	*trnT(ugu)*	*trnT(ugu)*	*trnT(ggu)*	*trnT(ugu)*	*trnT(ggu)*
trnT(ggu)	*trnT(ggu)*	—	*trnT(ugu)*	—	*trnT(ugu)*
trnV(uac)	*trnV(uac)*	*trnV(uac)*	*trnV(uac)*	*trnV(uac)*	*trnV(uac)*
—	*trnV(uac)*	—	—	—	—
trnW(cca)	*trnW(cca)*	*trnW(cca)*	*trnW(cca)*	*trnW(cca)*	*trnW(cca)*
trnY(gua)	*trnY(gua)*	*trnY(gua)*	*trnY(gua)*	*trnY(gua)*	*trnY(gua)*
tufA	*tufA*	*tufA*	*tufA*	*tufA*	*tufA*
ycf1	*ycf1*	*ycf1*	*ycf1*	*ycf1*	*ycf1*
ycf12	*ycf12*	—	*ycf12*	—	*ycf12*
ycf20	*ycf20*	—	*ycf20*	*ycf20*	*ycf20*
ycf3	*ycf3*	—	*ycf3*	*ycf3*	*ycf3*
ycf4	*ycf4*	—	*ycf4*	*ycf4*	*ycf4*
ycf47	*ycf47*	—	*ycf47*	—	*ycf47*

Genes in the inverted repeat are shown once.
