# Supplementary material for: The GC-Rich Mitochondrial and Plastid Genomes of the Green Alga Coccomyxa Give Insight into the Evolution of Organelle DNA Nucleotide Landscape
Source: PLoS One. 2011 Aug 26;6(8):e23624. doi: 10.1371/journal.pone.0023624 (PMC3162594; doi:10.1371/journal.pone.0023624)
Supplement: Table S3 — Amino acid composition of proteins encoded in complete mitochondrial genome sequences from trebouxiophytes (PDF) [file pone.0023624.s004.pdf]

Amino acid composition of proteins encoded in complete mitochondrial genomes from trebouxiophytes				
**Amino acid**	**Coccomyxa C-169**	**Prototheca wickerhamii**	**Helicosporidium sp.**	**Pedinomonas minor**
A	7.70%	6.60%	6.10%	4.60%
C	1.10%	1.50%	1.20%	0.70%
D	2.90%	3.20%	3.00%	2.30%
E	4.00%	3.40%	3.50%	2.00%
F	6.40%	6.90%	7.40%	13.60%
G	7.40%	5.80%	5.40%	5.50%
H	2.10%	2.00%	2.10%	1.40%
I	6.60%	8.60%	9.50%	11.80%
K	4.00%	5.80%	6.20%	2.50%
L	13.30%	11.80%	13.00%	16.60%
M	2.60%	2.50%	2.50%	1.50%
N	2.70%	4.80%	5.50%	4.90%
P	4.50%	3.60%	3.60%	2.60%
Q	3.40%	3.20%	3.20%	1.50%
R	5.60%	3.80%	3.40%	1.70%
S	8.00%	8.30%	7.50%	8.60%
T	4.80%	4.80%	5.00%	4.00%
V	7.40%	7.00%	5.40%	7.30%
W	1.70%	1.40%	1.20%	1.80%
Y	3.50%	4.60%	4.90%	4.90%
**GC content**	53.20%	25.80%	25.60%	22.20%
