# Supplementary material for: The GC-Rich Mitochondrial and Plastid Genomes of the Green Alga Coccomyxa Give Insight into the Evolution of Organelle DNA Nucleotide Landscape
Source: PLoS One. 2011 Aug 26;6(8):e23624. doi: 10.1371/journal.pone.0023624 (PMC3162594; doi:10.1371/journal.pone.0023624)
Supplement: Table S4 — Amino acid composition of proteins encoded in complete plastid genome sequences from trebouxiophytes (PDF) [file pone.0023624.s005.pdf]

Amino acid composition of proteins encoded in complete plastid genomes from trebouxiophytes						
**Amino acid**	**Coccomyxa C-169**	**Pedinomonas minor**	**Chlorella variabilis**	**Parachlorella kessleri**	**Leptosira terrestris**	**Helicosporidium sp.**
A	8.40%	7.00%	6.60%	6.20%	5.70%	4.00%
C	0.90%	0.80%	0.90%	1.00%	1.00%	0.90%
D	4.00%	3.70%	3.80%	3.60%	3.50%	3.30%
E	5.40%	5.60%	5.50%	5.00%	4.50%	4.90%
F	4.60%	5.80%	6.00%	6.20%	5.80%	6.80%
G	8.30%	6.80%	6.90%	6.70%	5.70%	4.50%
H	2.10%	1.90%	1.90%	2.00%	2.10%	1.70%
I	5.50%	6.60%	6.70%	7.60%	7.90%	10.20%
K	4.40%	5.70%	6.40%	6.70%	7.80%	10.80%
L	10.60%	11.20%	10.90%	10.50%	10.60%	11.30%
M	2.10%	2.00%	1.90%	1.80%	1.90%	1.50%
N	3.50%	4.60%	4.20%	5.30%	6.40%	8.00%
P	5.30%	4.20%	4.40%	4.20%	4.10%	3.50%
Q	4.40%	4.40%	4.60%	4.40%	4.80%	3.00%
R	6.40%	5.00%	5.30%	4.80%	4.60%	3.90%
S	7.10%	7.10%	6.70%	6.80%	6.90%	7.30%
T	5.50%	5.90%	5.90%	5.70%	5.80%	4.80%
V	7.00%	6.70%	6.60%	6.20%	5.50%	3.60%
W	1.50%	1.50%	1.50%	1.50%	1.20%	0.90%
Y	2.60%	3.00%	3.00%	3.30%	3.70%	4.90%
**GC content**	50.70%	34.80%	34.00%	30.00%	27.30%	26.90%
